# Molecular Techniques for Dicistrovirus Detection without RNA Extraction or Purification

**DOI:** 10.1155/2013/218593

**Published:** 2013-04-04

**Authors:** Jailson F. B. Querido, Jon Agirre, Gerardo A. Marti, Diego M. A. Guérin, Marcelo Sousa Silva

**Affiliations:** ^1^Centre for Malaria and Tropical Diseases, Instituto de Higiene e Medicina Tropical, Universidade Nova de Lisboa, Rua da Junqueira, 100, 1349-008 Lisboa, Portugal; ^2^Unidad de Biofísica (UBF), CSIC-UPV/EHU, Barrio Sarriena S/N, Bizkaia, 48940 Leioa, Spain; ^3^Centro de Estudios Parasitológicos y de Vectores (CEPAVE), CCT-La Plata/CONICET-UNLP, No. 584, 1900 La Plata, Argentina

## Abstract

Dicistroviridae is a new family of small, nonenveloped, and +ssRNA viruses pathogenic to both beneficial arthropods and insect pests as well. Triatoma virus (TrV), a dicistrovirus, is a pathogen of *Triatoma infestans* (Hemiptera: Reduviidae), one of the main vectors of Chagas disease. In this work, we report a single-step method to identify TrV, a dicistrovirus, isolated from fecal samples of triatomines. The identification method proved to be quite sensitive, even without the extraction and purification of RNA virus.

## 1. Introduction 

Triatoma virus (TrV), together with *Solenopsis invicta *virus-1, is one of the two members of the Dicistroviridae family pathogenic to insects of medical importance [1]. Because of its horizontal way of transmission and high pathogenicity in populations of triatomines, TrV has been postulated to be used as a biological control agent for both domiciliated and nondomiciliated vectors of Chagas disease. It replicates within the cytoplasm of gut cells of triatomines [2], causing a high mortality rate, delayed development, and a reduction in the fecundity [3, 4]. Recently, we have shown that this virus is unable to replicate nor is infective in mice and probably is not infective to other vertebrates, reinforcing the possibility of this virus to be used as a tool in biological control of triatomines transmitters of Chagas disease [5].

Dicistroviruses are characterized for having two open reading frames, ORF1 and ORF2. The ORF2 is responsible for encoding the structural proteins VP1, VP2, VP3, and VP4, which in the case of TrV have respective masses of 29.7 kDa, 28.4 kDa, 31.8 kDa, and 5.5 kDa [6]. The viral genome of TrV is a +ssRNA molecule of 9010 nucleotides [6]. 

A reliable method for the detection of TrV by molecular techniques represents an important step towards the identification of insect species being affected by this virus and the analysis of their geographical distribution as well. However, the diagnosis of triatomines in regions where it is difficult to conduct scientific investigation poses a serious challenge. In this study, we present a new method that allows the reduction of costs and time required for the entire process of diagnosis, enabling the detection of TrV without resorting to RNA extraction, and without running the risk of degrading the RNA during the extraction, purification and storage process.

## 2. Materials and Methods

### 2.1. Triatoma Virus Purification

The virus purification was made according to [6, 7]; dry feces coming from infected insects were collected on Whatman filter papers and excised from them using a standard surgery scalpel until 2 g of material was collected. A buffer composed of 10 mM NaCl, 1 mM MgCl_2_, and 200 mM citric acid at pH 6 (Lysis buffer) was added at a ratio of 100 mL per gram of dry material. PMSF (N 98.5%, purchased from Sigma) thawed at −20°C in isopropanol (Merck, GR for analysis) was added up to a final concentration of 1 mM. The mixture was homogenized for 5 min using a vortex mixer and then sonicated using a Sanyo MSE Soniprep 150 operating at 10 s ON/10 s OFF for 20 pulses. The homogenate was thereafter centrifuged in a Kontron T-1170 ultracentrifuge for 45 min at 20,000 g_avg_ using a Beckman SW28.1 rotor. The collected supernatant was then centrifuged for 2.5 h at 180,000 g_avg_ using a Kontron TFT50.38 rotor. The resulting pellet was resuspended overnight in 2 mL of 10 mM NaCl, 1 mM MgCl_2_, and 50 mM Tris at pH 7.4 (NMT buffer) and then loaded on top of a 35 mL continuous 5%–30% sucrose gradient. The centrifugation was at 100,000 g_avg_ for 3 h in a Beckman SW28.1 rotor. The gradient was fractionated from the bottom to the upper part in 0.5 mL aliquots that were collected in Greiner 96-well MasterBlocks and sealed thereafter with a silicone-based lid. The gradients were cooled in ice to avoid particle diffusion within them. The individual optical density of each aliquot was then measured at both 260 nm and 280 nm using an Implen NanoPhotometer coupled with an LG100 UV-G6786 cell. A baseline correction was performed using equivalent fractions from a control sucrose gradient. After plotting the absorbance profile, selected aliquots were loaded onto 12.5% polyacrylamide SDS-PAGE gels to check the purity of the samples. Following gradient fractionation, equivalent samples were pooled together and dialyzed overnight against 2 L of NMT buffer.

### 2.2. Reverse Transcriptase-PCR

For the RT-PCR, we use different concentration of purified TrV and feces samples (66.7 mg/mL), without resorting to RNA extraction. Dry feces coming from infected insects were resuspended in a buffer composed of 10 mM NaCl, 1 mM MgCl_2_, and 200 mM citric acid at pH 6 (Lysis buffer). PMSF (N 98.5%, purchased from Sigma) thawed at −20°C in isopropanol (Merck, GR for analysis) was added up to a final concentration of 1 mM. The homogenate was centrifuged in an Eppendorf centrifuge 5417 R for 30 min at 20,000 g_avg_) and was subsequently filtered through a 0.2 *μ*m. The feces sample was diluted in DEPC-treated water to a final concentration of 33.3 mg/mL. As a positive control, we used viral RNA extracted (Trizol, Invitrogen) from purified TrV and feces samples. The cDNA synthesis was performed according to cDNA Synthesis Kit Protocol (M-MuLV Reverse Transcriptase, Thermo Scientific). In the PCR reactions were used single primers pairs: TrV sense: 5′-TCAAAACTAACTATCATTCTGG-3′ (nt 7427 to 7448 in TrV ORF2 sequence) and TrV antisense: 5′-TTCAGCCTTATTCCCCCCC-3′ (nt 8240–8258 in TrV ORF2 sequence), with an expected product of 832 bp [8], selected from ORG2 region, conserved in dicistrovirus. PCR products were visualized on 1.5% agarose gels (running for 1 h at 100 V) stained with ethidium bromide, and their sizes were determined by comparison against DNA markers, HyperLadder I (Bioline, UK).

## 3. Results and Discussion

**Figure 1:**
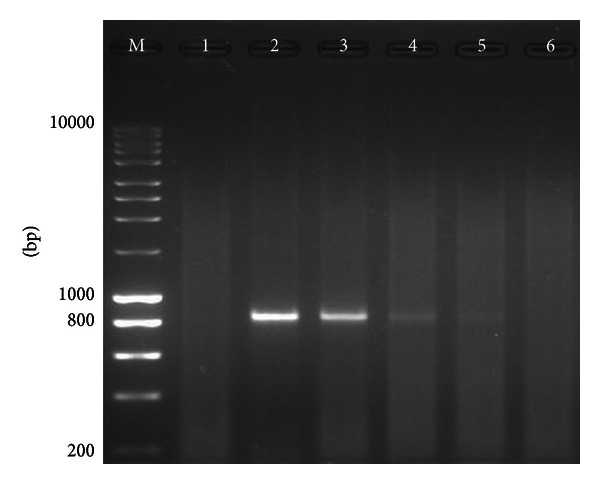
PCR products on 1,5% agarose gel stained with ethidium bromide. M: molecular weight marker 200 bp to 10000 bp (Bioline, UK). Lane 1: negative control, lane 2: 3000 ng of purified TrV, lane 3: 300 ng of purified TrV, lane 4: 30 ng of purified TrV, lane 5: 3 ng of purified TrV, and lane 6: 0,3 ng of purified TrV.

To obtain the cDNA, we used full genome TrV particles without RNA extraction. We used a concentration range between 3,000 and 0.3 ng of virus, being this concentration measurement based on the viral proteins, diluted in DEPC-treated water. The cDNA amplifications were performed using the selected primers pairs mentioned above. We obtained amplification until a minimum concentration of 3 ng of viral particles (Figure 1).

**Figure 2:**
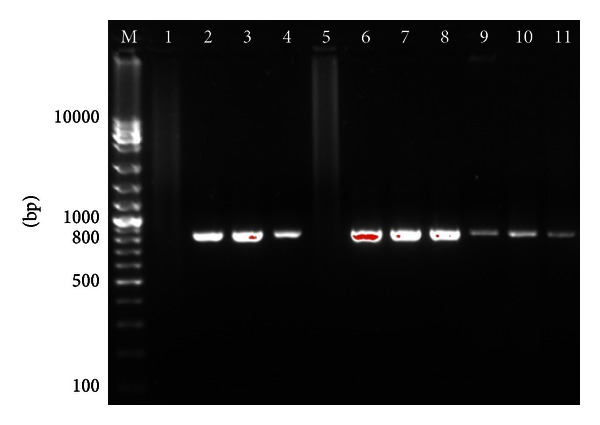
PCR products on 1,5% agarose gel. M: molecular weight marker 100 bp to 10000 bp (Jena Bioscience, Germany). Lane 1: negative control, lane 2: 5 *μ*L of viral RNA (from 3000 ng of purified TrV), lane 3: 5 *μ*L of viral RNA (from 300 ng of purified TrV), lane 4: 5 *µ*L of viral RNA (from 30 ng of purified TrV), lane 5: 5 *μ*L of viral RNA (from 0,03 ng of purified TrV), lane 6: 5 *μ*L of viral RNA (from 1 mg of feces), lane 7: 5 *μ*L of viral RNA (from 0.5 mg of feces), lane 8: 5 *μ*L of viral RNA (from 0,25 mg of feces), lane 9: 5 *μ*L of feces samples (without RNA extraction), lane 10: 3 *μ*L of feces samples (without RNA extraction), and lane 11: 1 *μ*L of feces samples (without RNA extraction).

To initiate the cDNA synthesis, we mixed 5 *µ*L of viral samples (5, 3, and 1 *μ*L for feces samples) with 100 pmol of Oligo (dT), incubated first at 65°C for 5 min and then at 4°C for 5 min. During the contact with the host cells, the virus released the genome. *In vitro*, this process may occur by varying certain environmental conditions such as pH or temperature [8, 9]. Incubating the virus at a temperature above 65°C will cause RNA release [8]. However, such temperature and time of incubation should not be too long because 65°C is close to the melting temperature of RNA. The results indicate that viral RNA can be detected in feces samples, after a single centrifugation and filtration step (Figure 2). These results reinforce the applicability and reproducibility of the method presented in the previous paragraph.

When we compare the result of RT-PCR from the samples analysed directly without resorting to RNA extraction to the samples where RNA extraction was proceeded, we can see that there is no significant difference between these two methods, which indicates that the method proposed here is a good alternative to the method traditionally used.

## 4. Conclusions

The method proposed in this study allows the detection of dicistrovirus by RT-PCR without resorting to RNA extraction, which reduces the cost associated with this method and the risk of RNA degradation during the process of RNA extraction, and it is swimmingly reproducible in a low-budget laboratory. Moreover, this study provides new facts about the thermostability of the TrV structure.
